# Predictors of Acceptance of Smartphone Application among People with Hypertension in India: A Cross-Sectional Study

**DOI:** 10.12688/wellcomeopenres.26425.1

**Published:** 2026-07-16

**Authors:** Vidhi Thakar, Sureshkumar Kamalakannan, V Prakash

**Affiliations:** 1 Ashok & Rita Patel Institute of Physiotherapy, Charotar University of Science and Technology, Changa, Gujarat, 388421, India; 2 Department of Social Work Education and Community Wellbeing, Northumbria University, Newcastle upon Tyne, England, NE1 8ST, UK; 3 Department of Neurophysiotherapy, MGM Institute of Physiotherapy, Chhatrapati Sambhaji Nagar, Maharashtra, 431003, India

**Keywords:** Hypertension, m-health, smartphone application, predictors, self-care

## Abstract

**Background:**

In India, nearly one-fourth of the population has hypertension, and only 17.5% of them have their blood pressure under control. Utilizing a smartphone application for hypertension care could potentially address the unmet needs within the existing healthcare delivery system. However, individuals with hypertension are the primary stakeholders for the implementation of such smartphone application-based care. Therefore, identifying the factors that influence the acceptance of a smartphone application-based care among individuals with hypertension is a prerequisite.

**Aim:**

This study aimed to identify factors influencing acceptance of smartphone application among people with hypertension using the extended Unified Theory of Acceptance and Use of Technology (UTAUT) model with age, gender, education, digital health literacy, m-health experience, status of blood pressure and its risk factors.

**Method:**

In this cross-sectional survey, 134 individuals with hypertension, aged 30–79 years, were recruited from public and private healthcare facilities located in both urban and rural areas. All participants completed the self-administered questionnaire.

**Results:**

Individuals who perceived the application to be easy to use were 9.79 (95% CI: 2.45–39.16, p - 0.001) times, and those who had the necessary technical resources and knowledge were 5.6 (95% CI: 1.86–16.80, p - 0.002) times more likely to accept smartphone application-based care. Other variables were either insignificant or had negligible influence on smartphone application-based care.

**Conclusion:**

The availability of technical resources and knowledge, as well as ease of use, are significant predictors of smartphone application acceptance among individuals with hypertension.

**Implications:**

Developers and policy-makers should consider incorporating these factors in developing and disseminating smartphone application-based care among individuals with hypertension.

## Plain Language summary

The burden of hypertension is escalating globally. Approximately 75% of the global population with hypertension resides in low-to middle-income countries. Notably, India is home to 50% of the low-to middle-income countries’ population with uncontrolled hypertension. To address the burden of hypertension, various care delivery approaches beyond standard care have recently been employed in India. However, the majority of these approaches have been deployed in public healthcare facilities. Thus, limiting access to 80% of the population who seek care from private hospitals and incur out-of-pocket expenses. This highlights a need for a care-delivery approach that is accessible and scalable. Integrating smartphone technologies into care delivery practices presents a significant opportunity as two-thirds of the Indian population owns a smartphone and has internet access.

Nonetheless, the introduction of a new form of care delivery should be acceptable to the intended population. Hence, in this study, we identified the factors that influence acceptance of smartphone application-based care. We found that ease of use and availability of technical resources and knowledge significantly influenced the acceptance of the smartphone application among people with hypertension.

## Background

In the past three decades, the burden of hypertension (HTN) has doubled globally, causing almost half of the heart disease or stroke-related deaths ( NCD Risk Factor Collaboration (NCD-RisC), 2021; Jeemon et al., 2021). India is no exception to this, with nearly a quarter(24.2%) of its population having HTN. Among the Indian population with HTN, only 17.5% have their blood pressure under control ( Koya et al., 2022) despite the recommendation of essential HTN care in resource-constrained settings by the International Society of Hypertension ( Unger et al., 2020) and the World Heart Federation ( Jeemon et al., 2021).

Poor HTN control is due to individual-level, healthcare professionals, and system-level barriers to implementing essential HTN care in India ( Gupta et al., 2019). Recent hypertension control programs like the India Hypertension Control Initiative (IHCI) ( Kaur et al., 2024) and the mPower Heart project ( Ajay et al., 2016) offer potential models for implementation; however, they are limited to public healthcare facilities. In countries like India, most of the population depends upon healthcare delivery by private hospitals with out-of-pocket expenses ( Das et al., 2021).

In addition, the current rate of adherence to the antihypertensive treatment is variable and ranges from 10%–90% among the population with HTN ( Unger et al., 2020; Dalal et al., 2021). Furthermore, non- adherence underlying HTN is multifactorial at the patient and healthcare system level ( Unger et al., 2020). Therefore, key barriers associated with suboptimal blood pressure control are limited access and poor adherence to recommended care ( Koya et al., 2022). A systematic care delivery strategy that prioritizes the unmet needs of people with HTN may be required to address these barriers.

Smartphone technology has enormous potential to address these barriers and assist those suffering from HTN ( Unger et al., 2020) given that more than half of India’s population uses smartphones and has internet accessibility ( Telecom Regulatory Authority of India, 2022). Even the National Digital Health Mission(2020) of India has recognized smartphones as a critical tool for digital healthcare infrastructure and patients as the key stakeholders in smartphone application development. However, to create a successful smartphone application, it is crucial to comprehend the targeted population acceptance of the application while considering the country-specific requirements ( Smith et al., 2015).

Many theories were proposed to understand and predict the acceptance and use of smartphone technology for industry, commerce, and healthcare. Our study used one of the most well-known models: the Unified Theory of Acceptance and Use of Technology (UTAUT) ( Venkatesh et al., 2003). This model includes the following factors: 1) ease of use (effort expectancy, EE), 2) benefits (performance expectancy, PE) achieved by using a smartphone application, 3) belief of a person’s significant others related to their use of a smartphone application (social influence, SI), and 4) perception of a person related to the availability of the resources and support in using an application (facilitating conditions, FC) ( Venkatesh et al., 2003).

The appropriateness of the UTAUT model in predicting acceptance of technology in healthcare, however, is dubious, especially in low to middle-income nations ( Venkatesh et al., 2011). As a result, in healthcare studies, context-specific factors should be included to increase the predictability of the UTAUT model ( Ahlan & Ahmad, 2014).

The influence of age and gender on smartphone application acceptance needs to be investigated considering the wide range of age groups(30–79 years of age) and both men and women being affected by HTN ( Jeemon et al., 2021). Furthermore, most people who have HTN are not aware of its status or its risk factors ( Jeemon et al., 2021). Thus, an investigation is needed to determine the influence of blood pressure status and the number of risk factors on smartphone application acceptance. Additionally, skills and experience to navigate and analyse the information provided by the smartphone application is required to make an informed decision ( Taylor & Todd, 1995; Norman & Skinner, 2006). Hence, necessitating investigation to determine the influence of education, digital health literacy, and m-health experience on acceptance of the smartphone application.

Thus, this study’s objective was to identify factors influencing the acceptance of a smartphone application among people with hypertension using the extended Unified Theory of Acceptance and Use of Technology (UTAUT) model with age, gender, education, digital health literacy, m-health experience, status of blood pressure and its risk factors.

## Method

### Study design & setting

People with HTN were recruited from public and private healthcare facilities located in urban and rural areas of two districts of an Indian state in this cross-sectional study. The study is reported according to the Strengthening the Reporting of Observational Studies in Epidemiology (STROBE) ( Vandenbroucke et al., 2007). This study received approval from the Institutional Ethics Committee of the first author’s affiliated university.

### Eligibility criteria

*Inclusion criteria*

1. Participants of both genders, 30–79 years of age ( Jeemon et al., 2021).
2. Participants who were diagnosed with hypertension(≥ a year) by a physician.
3. Participants who own and use an Android-based smartphone(≥ a year).

*Exclusion criteria*

1. Participants who had malignant hypertension, gestational hypertension or learning disability.
2. Participants who cannot read and understand English and/or Gujarati vernacular.

### Sample size

A priori power analysis was performed using G*Power version 3.1.9.7. Logistic regression was applied to estimate the required sample size to achieve 80% statistical power at a 5% significance level, considering an increase in odds of 1.67 among individuals with three or more hypertension risk factors as reported in a previous study ( Cherfan et al., 2020). The calculated minimum sample size was 111. To account for covariate effects at 0.04 and an anticipated 20% dropout rate due to potential non-completion of questionnaires, a convenience sample of 134 individuals with hypertension was recruited between February 2023 and December 2023.

### Procedure

**Figure 1.**
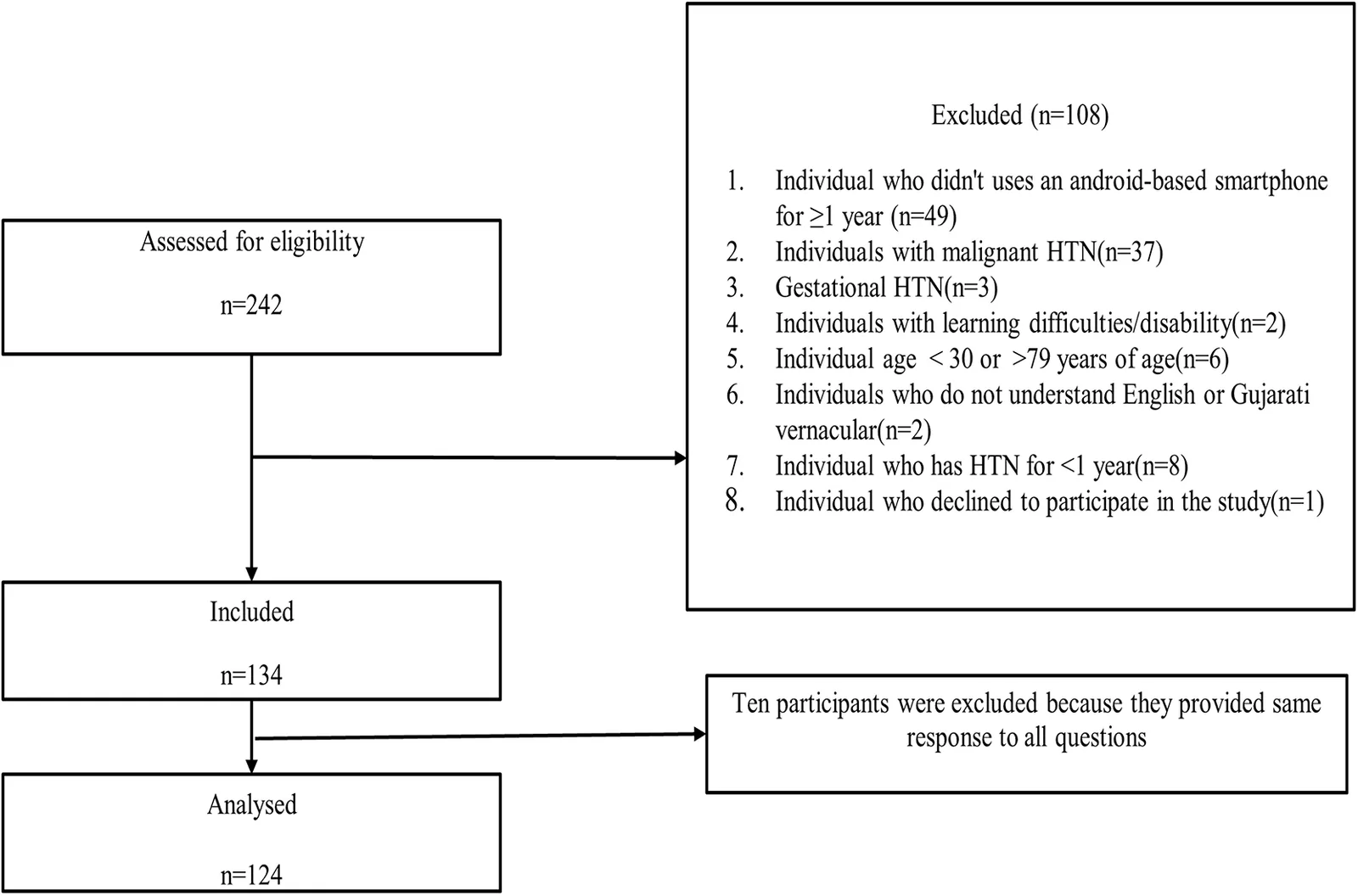
Study flow diagram.

A Physiotherapist(s) with a bachelor’s degree in Physiotherapy and a minimum of one year of experience in managing people with hypertension collected the data. Three Physiotherapists received training in data collection from the principal investigator. Eligible people received a letter detailing the study and a notification that they would be contacted to discuss participation. Figure 1 shows the study flow diagram. The data collection occurred face-to-face with the participants. At recruitment, the eligible person’s written informed consent was obtained. A blood pressure measurement was carried out using a sphygmomanometer, following the recommendation of the International Society of Hypertension ( Unger et al., 2020).

We collected data on age, gender, education, risk factors of hypertension, access to the internet, prescribed medicine & adherence to it, preferred type of exercise, day and time to exercise. All the questions measuring the dependent variable (acceptance of smartphone application) and predictor variables (digital health literacy (EHL), facilitating conditions (FC), effort expectancy (EE), social influence (SI), and performance expectancy (PE)) adapted from the previously validated instruments ( **Supplementary Table 1**). The EHL questionnaire exhibits moderate test-retest reliability (intraclass correlation r = 0.64) and excellent internal consistency (Cronbach’s α = 0.90) ( Norman & Skinner, 2006). All other constructs (Effort expectancy (EE), social influence (SI), performance expectancy (PE), and acceptance) exhibited satisfactory indicator reliability (ranging from 0.710 to 0.976), composite reliability (ranging from 0.822 to 0.935), and average variance extracted (AVE) that exceeded the 0.50 threshold ( Dou et al., 2017).

All variables were measured on a 5-point Likert scale ranging from 1, strongly disagree, to 5, strongly agree. The response was recorded in three categories for each item: “agree” (score of 4–5), “undecided” (score of 3), and “disagree” (score of 1–2) ( Rojanasumapong et al., 2021). The interpretation of the dependent variable- acceptance and predictor variable- effort expectancy was as follows: 1) High (agreed to at least 3/5 questions) and 2) Low (agreed to less than 3/5 questions). Social influence and Facilitating conditions were defined as follows: 1) High (agreed to at least 2/3 questions) and 2) Low (agreed to less than 2/3 questions). Performance expectancy classifies as 1) high (agreed to ≥3/4 questions) or 2) low (agreed to <3/4 questions) ( Rojanasumapong et al., 2021). Digital health literacy is interpreted as high for a score of ≥26 out of 40 & low for a score of <26 out of 40( Richtering et al., 2017).

Originally written in English, the master scale was translated into an Indian language (Gujarati) and field-tested. The questionnaire satisfied the independent English to Gujarati translation and back translation requirements before being administered ( World Health Organization, 2010). All participants completed the self-administered questionnaire.

### Statistical analysis

Data were analyzed using Statistical Package for Social Sciences software (SPSS), 23rd edition, IBM, United States. A predefined data dictionary was created for data entry. The data validity was assessed by the proofreading method ( Thakar et al., 2023). Before the analyses, the data were checked for completeness and coded. The mean and standard deviation were used to describe the continuous data. Percentages and frequencies were used for the categorical data. The dependent (acceptance of a smartphone application) and independent variables (age, gender, education, number of risk factors, facilitating conditions, effort expectancy, social influence, and performance expectancy) were categorical. Predictors for acceptance of smartphone-based care were determined using logistic regression tests. The magnitude of the relationship between dependent and predictor variables was explained using the odds ratio (OR) and 95% confidence interval (CI) at a 5% significance level.

## Results

All analyses were performed on the pooled dataset from all centers. Our study participants were relatively young (mean age 62(SD 10.2 years) and had an equal gender distribution (female, 68, 54.8%). Most of our participants had at least received primary school-based education(80, 64.5%), as shown in Table 1.

**Table 1.** Sociodemographic characteristics of participants.

Characteristics	Category	Frequency (n = 124)	Percentage(%)
Age (years)	<65	72	58.1
	≥65	52	41.9
Gender	Male	56	45.2
	Female	68	54.8
Education	College based	44	35.5
	School based	80	64.5
Location	Urban	66	53.2
	Rural	58	46.8
m-health use	Used	27	21.8
	Not used	97	78.2

All participants had access to the internet and were owners of Android-based smartphones. SMS and other messengers(110, 88.7%), music and audio(94, 75.8%), videos(80, 64.5%) were the features of the smartphones commonly used by the participants. Email(101, 81.5%), website(89, 71.8%) and health applications(97, 78.2%) were not used by our participants. The mean digital health literacy was 24(SD 7.5).

Each participant received a prescription for medicine; among them, 83(66.9%) received a prescription for monotherapy. One hundred and nine(87.9%) participants stated complete adherence to prescribed medicines. Participants also had various risk factors associated with hypertension. Seventy six participants(61.3%) were overweight/obese, 53(42.7%) were diabetic, 25(20.2%) consumed tobacco, and 4(3.2%) consumed alcohol occasionally, as shown in Table 2. One hundred and fourteen participants preferred to adopt the walking program because of hypertension and its risk factors. They didn’t have any preference for the day of the week, but they did like to choose the time of the day when to go for walks.

**Table 2.** Hypertension related characteristics of participants.

Characteristics	Category	Frequency (n = 124)	Percentage(%)
BMI (kg/m ^2^)	>30	20	16.1
	25 to 29.9	56	45.2
	18.5 to 24.9	46	37.1
	<18.5	2	1.6
Diabetes	Have	53	42.7
	Do not have	71	57.3
Alcohol consumption	Heavy drinking	0	0
	Drank in the past	3	2.4
	Light-moderate drinking	4	3.2
	Never drinking	117	94.4
Tobacco consumption	Current	25	20.2
	Past	9	7.3
	Never	90	72.6
Number of risk factors	3 or more	13	10.5
	2	36	29
	1	58	46.8
	0	17	13.7
Medicine adherence(%)	100	109	87.9
	75	11	8.9
	50	1	0.8
	25	1	0.8
	0	2	1.6
Blood pressure control	Controlled	67	54
	Uncontrolled	57	46

The majority of the respondents(84.7%) of this study showed interest in using a smartphone application-based care for HTN. This study’s findings indicate that significant predictors of acceptance of smartphone application-based care were effort expectancy, facilitating conditions, digital health literacy, and blood pressure. There was insufficient information (frequency in one of the cells was less than 5) to suggest that social influence was associated with acceptance of smartphone application-based care. Age, gender, education, m-health experience, number of risk factors, and performance expectancy were not associated significantly with acceptance of smartphone application-based care.

Table 3 shows the finding of the simple binary logistic regression. In this study, people with high facilitating conditions were 5.6(95%CI: 1.86–16.80, p-0.002) times more likely to accept application-based care. People who perceived that smartphone applications would be easy to use (High effort expectancy) were 13.97(95% CI: 3.78–51.55, p- < 0.001) times more likely to accept smartphone application-based care as compared to those who perceived application would be troublesome to use. Moreover, the odds of accepting application-based care increased by 1.06(95%CI: 1.01–1.10, p-0.012), 1.07(95%CI: 1.001–1.14, p-0.047), and 1.11 times(95%CI: 1.03–1.19, p-0.004) respectively; among those with high systolic and diastolic blood pressure, as well as those with high digital health literacy.

**Table 3.** Predictors of the acceptance of smartphone application-based care.

Predictors	B	SE for B	OR	p-value	95% CI	
					LL	UL
Facilitating conditions	1.72	0.56	5.60	0.002	1.866	16.803
Digital health literacy	0.11	0.04	1.11	0.004	1.034	1.192
Effort Expectancy	2.63	0.66	13.97	<0.001	3.789	51.554
Systolic blood pressure	0.06	0.02	1.06	0.012	1.012	1.107
Diastolic blood pressure	0.07	0.03	1.07	0.047	1.001	1.139

Table 4 shows the strength of the association of the predictor with acceptance of smartphone application-based care. Multivariate logistic regression was performed to find the most significant predictors among different factors influencing acceptance of application-based care. The 2-step model established that effort expectancy was the most significant predictor (aOR = 9.79, 95%CI: 2.45–39.16, p-0.001). The odds of accepting smartphone application-based care increased by 9.79 times for individuals who perceived that the applications would be user-friendly, considering their access to technical expertise and resources.

**Table 4.** Multivariate logistic regression.

Predictors	B	SE for B	OR	p-value	95% CI		R ^2^
					LL	UL	
Step-1							28.4
Constant	−2.04	0.86					
Effort Expectancy	2.63	0.66	13.97	<0.001	3.789	51.554	
Step-2							30.7
Constant	−2.79	1.03					
Effort Expectancy	2.82	0.71	9.79	0.001	2.449	39.163	
Facilitating conditions	0.86	0.63	2.37	0.169	0.693	8.090	

## Discussion

This study aimed to identify key factors influencing the acceptance of smartphone application-based care among individuals with hypertension. Univariate and multivariate analyses in this study revealed that effort expectancy was the most significant predictor of acceptance. We found that individuals with hypertension were more likely to accept application-based care if they perceived it to be easy to use. Our study finding is consistent with the findings of the study by Alalwan et al. (2017). They also found effort expectancy to be a significant predictor of acceptance.

Facilitating conditions was another important construct influencing the acceptance of smartphone application-based care in this study. Similar findings were obtained in the study conducted in Bangladesh ( Alam et al., 2020). We did not find performance expectancy as a significant predictor despite prior research suggesting its impact on the acceptance of care. We attribute this finding to the participant’s perception that smartphone-based care will offer no advantage over in-person consultations with healthcare professionals. We also had inconclusive results related to social influence, as fewer than five persons represented both the characteristics of “having social support” and “not being interested in the future acceptance of smartphone-based care”.

We found poor digital health literacy among our participants. These findings are consistent with the findings of the studies conducted in Japan ( Mitsutake et al., 2016), whereas the study conducted in Thailand reported higher digital health literacy ( Rojanasumapong et al., 2021). Comparing the scores among different countries’ populations may not be appropriate here, considering they have different demographics, socioeconomic status, internet availability, and accessibility. Furthermore, digital health literacy had negligible influence, and the m-health experience did not influence the acceptance of smartphone application-based care. We believe this occurred because individuals with hypertension expected to receive training for this newer form of care.

We did not find age, gender, and education influencing the acceptance of smartphone application-based care, even though previous studies reported it ( Norman & Skinner, 2006; Zhao et al., 2018). Smartphone application-based care is still in its early stages in India; therefore, we believe we did not obtain any difference in acceptance between age groups, levels of education, and genders.

The odds ratio was close to 1 for blood pressure, which is suggestive of the negligible influence of it on the acceptance of application-based care ( Di Lorenzo et al., 2014). We also did not find the impact of multiple risk factors on the acceptance of application-based care. One of the possible contributing factors to this may be participants’ lack of awareness about hypertension and their status of blood pressure control ( Jeemon et al., 2021). All participants in this study received a medication prescription consistent with the recommendations of the International Society of Hypertension ( Unger et al., 2020). Despite this, uncontrolled hypertension was prevalent in 46% participants, 12.1% had poor adherence to the prescribed medication, and 86.3% had risk factors for hypertension.

This occurrence is a result of the current care delivery system. They are focused on pharmacological intervention, even though exercise and medicine are equally effective in reducing blood pressure ( Naci et al., 2019). Therefore, to include additional essential hypertension management domains in future application-based care, participants in this study were asked about their preferred exercise behavior. The majority of the participants preferred to adopt a walking program(91.9%) and showed interest in using smartphone application-based care in the future (84.7%).

The identified factors in this study are the preferences of people residing in peculiar socio-cultural and geographical locations. Therefore, the characteristics of the participants in this study provide information about who will be eligible to use the novel smartphone application-based care. In this study, participants were recruited from urban and rural areas who had access to the internet. All participants were Android-based smartphone users and have used SMS, audio, and video features of the smartphone.

### Strengths and limitations

Among the several strengths, this study includes representative populations from urban and rural areas, public and private healthcare facilities, those having controlled and uncontrolled hypertension, and those who adhere or did not adhere to the prescribed medication. Thus increasing the generalizability of the findings of this study across populations with HTN in resource-constrained settings. Moreover, most participants had not utilized smartphones for health-related activities, highlighting the necessity for creating smartphone-based hypertension management programs.

Our study has a few limitations. The length of the questionnaire prevented us from further investigating other factors like perceived risk, trust, etc. Univariate analysis yielded a wider confidence interval, while multivariate analysis, which adjusted for another factor, offered a credible confidence interval for effort expectancy in this investigation. Social desirability bias may have contributed to most of the participants’ expression of interest in using the smartphone application in the future.

### Implications

This study identified two antecedent factors integral to the acceptance of a smartphone application for hypertension: effort expectancy and facilitating conditions. Thus, while creating and commissioning smartphone application-based programs to address self-care among people with hypertension, app developers and policymakers may focus on making the application convenient and easy for users to adopt. The developers may also be mindful of the technological capability of the region where they intend to provide care and the person’s technical knowledge, thereby ascertaining the compatibility of the devised care program with a person with hypertension.

The development of smartphone applications considering the requirements of the person with hypertension may play a pivotal role in improving access to health-related information and services for people with hypertension and load-sharing for healthcare professionals. In addition, evidence-based information and recommendations via smartphone applications in the future may contribute to secondary prevention in chronic conditions like hypertension.

## Conclusion

This study provided insight into the factors influencing the acceptance of smartphone application-based care among people with hypertension. Ease of use (effort expectancy), accessibility, and availability of technical knowledge and resources (facilitating conditions) were the factors influencing acceptance of smartphone application-based care among people with hypertension.

## Ethics approval

This study received approval from the Institutional Ethics Committee of the first author’s affiliated university (CHA/IEC/ADM/23/04/410).

## Data Availability

Figshare: Factors influencing acceptance of smartphone application among people with hypertension, **https://doi.org/10.6084/m9.figshare.31923069** ( Thakar et al., 2026). This project contains the following extended data: **Supplementary Table 1.** Measurement items and participants’ responses in percentage. Data are available under the terms of the Creative Commons Attribution 4.0 International license (CC-BY 4.0).
