# Supplementary material for: Predictors of Acceptance of Smartphone Application among People with Hypertension in India: A Cross-Sectional Study
Source: Wellcome Open Res. 2026 Sep 9;11:450. Originally published 2026 Jul 16. [Version 3] doi: 10.12688/wellcomeopenres.26425.3 (PMC13616054; doi:10.12688/wellcomeopenres.26425.3)
Supplement: Peer review history

# Reviewer report: Reviewer response for version 3

**DOI:** 10.21956/wellcomeopenres.30474.r170139

**Published:** 2026-09-26

**Authors:** Prima Trisna Aji

**Affiliations:** 1 Universitas Muhammadiyah Semarang, Semarang, Central Java, Indonesia

This research article examines predictors of the acceptance of smartphone application-based care among people with hypertension in India using an extended Unified Theory of Acceptance and Use of Technology (UTAUT) framework. The topic is relevant to the growing implementation of digital health and mHealth approaches for chronic disease management, particularly hypertension, and the inclusion of participants from both urban and rural healthcare settings adds practical relevance to the study.

 Overall, the manuscript is clearly structured and the rationale for investigating technology acceptance among people with hypertension is adequately presented. The cross-sectional design is appropriate for examining associations between the proposed predictors and smartphone application acceptance. The authors provide sufficient information regarding the study setting, participant eligibility, recruitment, sample size estimation, measurement instruments, translation procedures, data collection, and statistical analysis to allow readers to understand and potentially replicate the study.

 The statistical analysis is presented transparently. Importantly, the authors distinguish the findings of the primary multivariable analysis from those of the sensitivity analysis. In the primary analysis, effort expectancy was significantly associated with acceptance of smartphone application-based hypertension care. In the sensitivity analysis, however, this association was attenuated and no longer statistically significant, whereas social influence emerged as a significant predictor. The manuscript appropriately acknowledges this difference and avoids presenting the primary finding as uniformly robust across analytical approaches. Given the relatively wide confidence intervals around some estimates, the cautious interpretation adopted in the revised manuscript is appropriate.

 The Discussion generally reflects the results and places the findings within the context of the UTAUT framework and previous digital health research. The authors also appropriately acknowledge relevant limitations, including the cross-sectional nature of the study, the modest sample size, the categorisation of UTAUT scores in the primary analysis, and the potential limitations regarding generalisability.

 The conclusion is adequately supported by the reported findings and appropriately distinguishes between the primary and sensitivity analyses. The recommendation that future studies confirm the independent contributions of technological, social, and individual factors is reasonable given the observed variability between analytical approaches.

 The availability of the underlying dataset and supplementary materials further supports transparency and reproducibility.

 Overall, the revised manuscript provides a useful contribution to understanding the acceptance of smartphone application-based hypertension care in the Indian context. The methodological and interpretative issues raised during the earlier review process appear to have been substantially addressed, and I have no major concerns that would prevent the article from being considered scientifically sound in its current version.

Is the work clearly and accurately presented and does it cite the current literature?

Yes

If applicable, is the statistical analysis and its interpretation appropriate?

Yes

Are all the source data underlying the results available to ensure full reproducibility?

Yes

Is the study design appropriate and is the work technically sound?

Yes

Are the conclusions drawn adequately supported by the results?

Yes

Are sufficient details of methods and analysis provided to allow replication by others?

Yes

Reviewer Expertise:

Medical-Surgical Nursing; Cardiovascular Nursing; Hypertension Management; Chronic Disease Self-Management; Digital Health and mHealth; Technology-Assisted Health Education; Community-Based Nursing; and Patient Self-Care.

I confirm that I have read this submission and believe that I have an appropriate level of expertise to confirm that it is of an acceptable scientific standard.

# Reviewer report: Reviewer response for version 3

**DOI:** 10.21956/wellcomeopenres.30474.r167551

**Published:** 2026-09-18

**Authors:** Saowaluck Sukpattanasrikul

**Affiliations:** 1 Mahidol University, Bangkok, Thailand

I am satisfied that the authors have adequately addressed the major concerns raised in the previous review rounds, and I consider the manuscript acceptable for indexation. I therefore approve the manuscript. However, I have only one minor editorial comment. Please revise the Plain Language Summary to ensure it is fully consistent with the sensitivity analysis and the main Conclusion, particularly regarding the findings on effort expectancy and social influence.

 No further statistical analysis is requested.

Is the work clearly and accurately presented and does it cite the current literature?

Partly

If applicable, is the statistical analysis and its interpretation appropriate?

Partly

Are all the source data underlying the results available to ensure full reproducibility?

No

Is the study design appropriate and is the work technically sound?

Partly

Are the conclusions drawn adequately supported by the results?

Partly

Are sufficient details of methods and analysis provided to allow replication by others?

Partly

Reviewer Expertise:

Gerontological Nursing                                                                  Non-Communicable Diseases Care, especially hypertension

I confirm that I have read this submission and believe that I have an appropriate level of expertise to confirm that it is of an acceptable scientific standard.

# Reviewer report: Reviewer response for version 2

**DOI:** 10.21956/wellcomeopenres.30313.r165510

**Published:** 2026-08-28

**Authors:** Saowaluck Sukpattanasrikul

**Affiliations:** 1 Mahidol University, Bangkok, Thailand

The revised manuscript addresses most of my previous comments. The rationale for using UTAUT, the definition of acceptance, the description of the instruments and translation process, the ethical approval date, the sensitivity analysis, and the reporting of crude and adjusted estimates are now clearly presented. The following comments remain for consideration.

 1. On page 5, in the Methods section, the manuscript clarifies that participants were recruited from one public and three private healthcare facilities. However, the procedure for selecting these facilities remains unclear. Please specify whether the facilities were selected purposively, by convenience, based on accessibility or institutional permission, or through another method, with rationale 

 2. My previous comment requested clarification of the variable-entry procedure, including the criteria for inclusion and removal. Although the Methods section now states that predictors with p≤0.05 in the univariable analysis were entered into the hierarchical model, the reported multivariable analysis does not align with this criterion. Table 3 identifies five predictors meeting the threshold, while Table 4 includes only effort expectancy and facilitating conditions. Please clarify why the remaining variables were excluded and justify the order of entry, or revise the analysis and Methods for consistency. Once the final multivariable model is confirmed, ensure that the Results, Discussion, and Conclusion sections are fully aligned with the final analysis.

 3. The limitations section should state that a formal psychometric evaluation of the Gujarati questionnaire was not conducted and should explicitly acknowledge the potential for self-report bias, in addition to the social-desirability bias already reported.

 4. Please reconsider the appropriateness of this term “representative populations”, given the use of convenience sampling and the restriction to existing Android users who could read English or Gujarati. Instead, state that the sample included participants from urban and rural areas and from both public and private healthcare facilities.

Is the work clearly and accurately presented and does it cite the current literature?

Partly

If applicable, is the statistical analysis and its interpretation appropriate?

Partly

Are all the source data underlying the results available to ensure full reproducibility?

No

Is the study design appropriate and is the work technically sound?

Partly

Are the conclusions drawn adequately supported by the results?

Partly

Are sufficient details of methods and analysis provided to allow replication by others?

Partly

Reviewer Expertise:

Gerontological Nursing                                                                  Non-Communicable Diseases Care, especially hypertension

I confirm that I have read this submission and believe that I have an appropriate level of expertise to confirm that it is of an acceptable scientific standard, however I have significant reservations, as outlined above.

# Response

**Published:** 2026-09-03

**Authors:** Vidhi Thakar

**Affiliations:** Ashok & Rita Patel Insititute of Physiotherapy, Charotar University of Science and Technology, Changa, Gujarat, India

Dear Reviewer. Thank you for your ongoing review. We have revised the manuscript based on your comments and believe we have adequately addressed the concerns raised.****

**Comments:**

 1. On page 5, in the Methods section, the manuscript clarifies that participants were recruited from one public and three private healthcare facilities. However, the procedure for selecting these facilities remains unclear. Please specify whether the facilities were selected purposively, by convenience, based on accessibility or institutional permission, or through another method, with rationale.

**Authors’ response:** We have elaborated the methods subsection to describe the procedure for selecting healthcare facilities for participant recruitment.

 2. My previous comment requested clarification of the variable-entry procedure, including the criteria for inclusion and removal. Although the Methods section now states that predictors with p≤0.05 in the univariable analysis were entered into the hierarchical model, the reported multivariable analysis does not align with this criterion. Table 3 identifies five predictors meeting the threshold, while Table 4 includes only effort expectancy and facilitating conditions. Please clarify why the remaining variables were excluded and justify the order of entry, or revise the analysis and Methods for consistency. Once the final multivariable model is confirmed, ensure that the Results, Discussion, and Conclusion sections are fully aligned with the final analysis.

**Authors’ response:** We thank the reviewer for identifying the need for clarity regarding the variable entry procedure. Significant predictors (p ≤ 0.05) were entered into the hierarchical logistic regression analysis in blocks based on their conceptual relevance to acceptance of the smartphone application. The first block consists of core Unified Theory of Acceptance and Use of Technology (UTAUT) predictors followed by contextual and finally clinical predictors. We have explicitly described the variable entry procedure in the Methods section and revised the Results section to ensure consistency with the final model.

 3. The limitations section should state that a formal psychometric evaluation of the Gujarati questionnaire was not conducted and should explicitly acknowledge the potential for self-report bias, in addition to the social-desirability bias already reported.

**Authors’ response: **We have expanded the limitations section. It now explicitly acknowledges the potential for self-reported bias and the lack of a formal psychometric evaluation of the Gujarati questionnaire

 4. Please reconsider the appropriateness of this term “representative populations”, given the use of convenience sampling and the restriction to existing Android users who could read English or Gujarati. Instead, state that the sample included participants from urban and rural areas and from both public and private healthcare facilities. 

**Authors’ response: **We agree with the reviewer. We have revised the wording in manuscript to appropriately describe the study sample and removed the term “representative populations”.

 Thank you for your input.

 Yours sincerely,

 Vidhi Thakar on behalf of the co-authors

# Reviewer report: Reviewer response for version 1

**DOI:** 10.21956/wellcomeopenres.29101.r161551

**Published:** 2026-08-05

**Authors:** Saowaluck Sukpattanasrikul

**Affiliations:** 1 Mahidol University, Bangkok, Thailand

This manuscript has potential value for understanding the predictors of smartphone application acceptance among people with hypertension in India. However, several aspects of the study require further clarification, as outlined below.

**Abstract**

 • In the Abstract, the Methods subsection should report that 134 participants completed the questionnaire, whereas the Results subsection should state that 124 participants were analyzed.

 • In the Abstract, the Results subsection currently mixes the unadjusted estimate for facilitating conditions (OR 5.60) with the adjusted estimate for effort expectancy (aOR 9.79). Please clearly separate unadjusted and adjusted findings. The main results should focus on the multivariable model, where effort expectancy stayed significantly linked to acceptance, but facilitating conditions did not. If you keep the unadjusted estimate for facilitating conditions, label it as a crude OR and report it apart from the adjusted results.

**Introduction**

 • In paragraph 5, please provide a clearer rationale for selecting UTAUT as the theoretical framework. Specifically, explain why it is appropriate for examining smartphone-based care in the study population and setting and why it was preferred over other technology-acceptance models.

 • In the paragraph where you introduce the UTAUT model, please clarify how you defined “acceptance” in this study compared to behavioral intention and use behavior in the original UTAUT framework. Say whether acceptance means willingness or intention to use a smartphone app, expected uptake, or actual use.

 • The knowledge gap needs to be clearer. Please briefly summarize what is already known about smartphone app acceptance among people with hypertension, point out the limits of earlier studies, and explain how your study addresses what is still unknown, especially in the Indian private healthcare setting and by using an extended UTAUT model.

**Method**

 • Page 4, the Methods section does not specify how many public versus private facilities were involved or how they were selected. Please provide further explanation. (No need to name the specific state, districts, or facilities.)

 • On page 5, the explanation for the sample size needs to be clearer. Since you used G*Power's logistic regression module, the value 0.04 for "covariate effects" may refer to R² or another parameter, but this is unclear. Please confirm what this value means and provide a citation or rationale for using a 20% dropout rate.

 • On page 5, besides reporting the validity and reliability of the instruments, please add more details about the tool used for each variable. Include the instrument name, who developed it, how many items it has, its domains or dimensions, how it is scored, and how to interpret the scores.

 • On page 5, responses on the 5-point scales were split into "high" and "low" for all variables using a cut-off from a Thai study. This approach loses some information and may not match the score distribution in your sample, which could affect the results. Please explain why you chose each cut-off for this sample and consider doing a sensitivity analysis for each variable by using the total or average score as a continuous variable to check whether the results remain the same.

 • On page 5, the manuscript mentions independent translation and back-translation and "field-testing" of the Gujarati scale but does not give details. Please say who did the translations, whether the back-translators did not see the original, what was done during field-testing, and whether you checked the reliability and validity of the Gujarati version after translation.

 • Page 6, the variable-entry procedure for the logistic regression analysis is not described in the Data Analysis section. Please specify the method used to enter variables into the model, the candidate predictors evaluated, and the statistical or theoretical criteria applied for variable inclusion and removal.

 • Figure 1 says that people who had not used an Android-based smartphone for at least one year were excluded, but the inclusion criteria say participants must have owned and used an Android-based smartphone for at least one year. Please clarify whether both ownership and use were required, and ensure the wording is consistent throughout the manuscript.

 • Ethics approval date unclear relative to data collection start. Page 10 gives the ethics approval reference as CHA/IEC/ADM/23/04/410, while page 5 states that data collection began in February 2023. Since the manuscript does not explicitly state the approval date, it isn't possible to confirm whether data collection began before or after approval was granted. Please clarify the exact approval date and confirm that no participants were enrolled before ethics approval.

**Results**

 ● Page 6, the manuscript describes participants as "relatively young," but the reported mean age is 62 years (SD 10.2, range 30–79) — not typically characterized as young. Please revise this description and consider whether the age distribution has implications for interpreting the findings. Similarly, the 45.2%/54.8% male-to-female split is described as "equal," though a nearly 10-point difference would be more accurately called comparable or roughly balanced.

 ● In Table 4, Step 2, the coefficient for Effort Expectancy (B = 2.82) does not match the reported OR of 9.79 and its 95% CI (2.449–39.163). An OR of 9.79 should correspond to a coefficient of about 2.28. Please check and correct the B value if needed.

**Discussion**

 • Page 9, revise the claim of representativeness and broad generalizability, like in resource-constrained settings, because the sample was selected from two districts and restricted to current Android users attending healthcare facilities who could read English or Gujarati.

 • Please expand the limitations section to address convenience sampling, restriction to existing smartphone users, self-report and social-desirability bias, and the dichotomization of continuous scales (noting the results of the requested sensitivity analysis, if performed).

**Other**

 • Please include a completed STROBE checklist, with the relevant manuscript page numbers, as supplementary material.

Is the work clearly and accurately presented and does it cite the current literature?

Partly

If applicable, is the statistical analysis and its interpretation appropriate?

Partly

Are all the source data underlying the results available to ensure full reproducibility?

No

Is the study design appropriate and is the work technically sound?

Partly

Are the conclusions drawn adequately supported by the results?

Partly

Are sufficient details of methods and analysis provided to allow replication by others?

Partly

Reviewer Expertise:

Gerontological Nursing                                                                  Non-Communicable Diseases Care, especially hypertension

I confirm that I have read this submission and believe that I have an appropriate level of expertise to confirm that it is of an acceptable scientific standard, however I have significant reservations, as outlined above.

# Response

**Published:** 2026-08-17

**Authors:** Vidhi Thakar

**Affiliations:** Ashok & Rita Patel Insititute of Physiotherapy, Charotar University of Science and Technology, Changa, Gujarat, India

***Reviewer 1:** This manuscript has potential value for understanding the predictors of smartphone application acceptance among people with hypertension in India. However, several aspects of the study require further clarification, as outlined below*

***Authors’ comment:** We appreciate the reviewer’s detailed and constructive feedback. We are glad to hear that this manuscript has potential value for understanding the predictors of smartphone application acceptance among people with hypertension in India.  We have responded to each comment below and revised the manuscript accordingly*

**Major comments:**

 1. In the Abstract, the Methods subsection should report that 134 participants completed the questionnaire, whereas the Results subsection should state that 124 participants were analyzed.

**Authors’ response:** We thank the reviewer for highlighting this discrepancy. We have revised in the abstract the method subsection which now states 134 participants completed the questionnaire while the result subsection reports that 124 participants were analyzed.

 2. In the Abstract, the Results subsection currently mixes the unadjusted estimate for facilitating conditions (OR 5.60) with the adjusted estimate for effort expectancy (aOR 9.79). Please clearly separate unadjusted and adjusted findings. The main results should focus on the multivariable model, where effort expectancy stayed significantly linked to acceptance, but facilitating conditions did not. If you keep the unadjusted estimate for facilitating conditions, label it as a crude OR and report it apart from the adjusted results.

**Authors’ response:** We agree with the reviewer. The abstract result subsection is revised and now the primary emphasis is placed on the multivariable model with reporting of adjusted estimates for effort expectancy.

 3. In paragraph 5, please provide a clearer rationale for selecting UTAUT as the theoretical framework. Specifically, explain why it is appropriate for examining smartphone-based care in the study population and setting and why it was preferred over other technology-acceptance models.

**Authors’ response:** Thank you for this suggestion. We have expanded the rationale for selecting the Unified Theory of Acceptance and Use of Technology (UTAUT). We have clarified that the UTAUT was selected for its ability to explain social, infrastructural, organizational, behavioural, and technological factors of technology acceptance and is not restricted to perceived benefits and ease of use as in the Technology acceptance model(TAM). Furthermore, it was extended to include factors such as digital health literacy which is relevant for smartphone application-based hypertension care.

 4. In the paragraph where you introduce the UTAUT model, please clarify how you defined “acceptance” in this study compared to behavioral intention and use behavior in the original UTAUT framework. Say whether acceptance means willingness or intention to use a smartphone app, expected uptake, or actual use.

**Authors’ response:** We agree with the reviewer that this requires clarification. In this study, acceptance means willingness or behavioral intention to use a smartphone application-based care rather than the actual use behaviour. The definition of acceptance is included in the introduction section.

 5. The knowledge gap needs to be clearer. Please briefly summarize what is already known about smartphone app acceptance among people with hypertension, point out the limits of earlier studies, and explain how your study addresses what is still unknown, especially in the Indian private healthcare setting and by using an extended UTAUT model. 

**Authors’ response:** We have expanded the introduction section to provide clearer knowledge gap. Previous studies on smartphone application-based hypertension care have identified perceived benefits, perceived usefulness, social influence as factors which influence acceptance of such technology. However, the majority of these studies were conducted outside India. In India, technology assisted hypertension care is available however there is limited evidence in regards to its acceptance and use among people with hypertension particularly in private healthcare settings.

 6. Page 4, the Methods section does not specify how many public versus private facilities were involved or how they were selected. Please provide further explanation. (No need to name the specific state, districts, or facilities.

**Authors’ response:** Thank you for your suggestion. We have included in the method section the number of public and private facilities involved in the present study.

 7. On page 5, the explanation for the sample size needs to be clearer. Since you used G*Power's logistic regression module, the value 0.04 for "covariate effects" may refer to R² or another parameter, but this is unclear. Please confirm what this value means and provide a citation or rationale for using a 20% dropout rate.

**Authors’ response:** Thank you for identifying this ambiguity. We have clarified the sample size calculation subsection. The value 0.04 represents R² of other X and citation is provide for using a 20% potential non-response rate.

 8. On page 5, besides reporting the validity and reliability of the instruments, please add more details about the tool used for each variable. Include the instrument name, who developed it, how many items it has, its domains or dimensions, how it is scored, and how to interpret the scores.

**Authors’ response:** We have expanded the details of the instruments (items, scoring and interpretation) used in the present study in the method section. The digital health literacy was assessed using the e-health Literacy scale developed by Norman and Skinner(2008). The technology acceptance constructs (effort expectancy, performance expectancy, social influence, acceptance) were adapted from Dou et al.(2017).

 9. On page 5, responses on the 5-point scales were split into "high" and "low" for all variables using a cut-off from a Thai study. This approach loses some information and may not match the score distribution in your sample, which could affect the results. Please explain why you chose each cut-off for this sample and consider doing a sensitivity analysis for each variable by using the total or average score as a continuous variable to check whether the results remain the same.

**Authors’ response:** We thank the reviewer for this important methodological observation. We agree that dichotomization of 5-point scales may result in loss of some information and might influence the strength and significance of associations. In the primary analysis we retained the original categorization because this methodological approach was used and reported by previously published studies. However, we agree that it may not match the score distribution of the sample in the present study. As suggested, we performed sensitivity analysis in which the predictors: effort expectancy, performance expectancy, facilitating conditions, social influence, digital health literacy were entered as continuous score and acceptance was retained as a binary outcome as it was in the primary analysis. We have included description of the sensitivity analysis in the data analysis and result section. We have discussed the discrepancy between primary and sensitivity analysis in the discussion section and acknowledge it in the limitation section. 

 10. On page 5, the manuscript mentions independent translation and back-translation and "field-testing" of the Gujarati scale but does not give details. Please say who did the translations, whether the back-translators did not see the original, what was done during field-testing, and whether you checked the reliability and validity of the Gujarati version after translation.

**Authors’ response:** We have provided the details of independent translation and back-translation of the scale in the method section. Two bilingual translators independently translated English scale into Gujarati and another 2 bilingual translators did the back-translation. The field testing was intended to examine the clarity and comprehension therefore psychometric evaluation was not done.

 11. Page 6, the variable-entry procedure for the logistic regression analysis is not described in the Data Analysis section. Please specify the method used to enter variables into the model, the candidate predictors evaluated, and the statistical or theoretical criteria applied for variable inclusion and removal.

**Authors’ response:** We have clarified variable entry procedure for the logistic regression analysis in the data analysis section.

 12. Figure 1 says that people who had not used an Android-based smartphone for at least one year were excluded, but the inclusion criteria say participants must have owned and used an Android-based smartphone for at least one year. Please clarify whether both ownership and use were required, and ensure the wording is consistent throughout the manuscript.

**Authors’ response:** Thank you for identifying this inconsistency. Use of Android-based smartphone for at least one year was required for participation. We have revised the inclusion criteria to ensure the wording is consistent throughout the manuscript.

 13. Ethics approval date unclear relative to data collection start. Page 10 gives the ethics approval reference as CHA/IEC/ADM/23/04/410, while page 5 states that data collection began in February 2023. Since the manuscript does not explicitly state the approval date, it isn't possible to confirm whether data collection began before or after approval was granted. Please clarify the exact approval date and confirm that no participants were enrolled before ethics approval.

**Authors’ response:** We thank the reviewer for identifying this important issue. The ethics approval for the present study was obtained on 19 ^th^ January 2023 with ethics approval reference: CHA/IEC/ADM/23/04/410. Therefore, ethics approval was obtained before beginning of participant recruitment in February 2023 and no participants were enrolled before ethics approval was granted. The subsequent request to the ethics committee was regarding the inclusion of the data collection sites names required for submitting an application to the Clinical Trial Registry of India. This was necessary because present study is part of a doctoral research.

 14. Page 6, the manuscript describes participants as "relatively young," but the reported mean age is 62 years (SD 10.2, range 30–79) — not typically characterized as young. Please revise this description and consider whether the age distribution has implications for interpreting the findings. Similarly, the 45.2%/54.8% male-to-female split is described as "equal," though a nearly 10-point difference would be more accurately called comparable or roughly balanced

**Authors’ response:** Thank you. We have revised the description of age and gender in the result section.

 15. In Table 4, Step 2, the coefficient for Effort Expectancy (B = 2.82) does not match the reported OR of 9.79 and its 95% CI (2.449–39.163). An OR of 9.79 should correspond to a coefficient of about 2.28. Please check and correct the B value if needed.

**Authors’ response:** Thank you for identifying this inconsistency. We have checked and corrected the B value in table 4.

 16. Page 9, revise the claim of representativeness and broad generalizability, like in resource-constrained settings, because the sample was selected from two districts and restricted to current Android users attending healthcare facilities who could read English or Gujarati.

**Authors’ response:** We have removed the claim of representativeness and broad generalizability from the strength and limitation subsection.

 17. Please expand the limitations section to address convenience sampling, restriction to existing smartphone users, self-report and social-desirability bias, and the dichotomization of continuous scales (noting the results of the requested sensitivity analysis, if performed).

**Authors’ response:** The point is well taken. We have expanded the limitation section and it addresses use of convenience sampling, restriction to existing smartphone users, self-report and social-desirability bias, and the dichotomization of continuous scales.

 18. Please include a completed STROBE checklist, with the relevant manuscript page numbers, as supplementary material.

**Authors’ response:** Thank you. We have completed STROBE checklist and have included it as supplementary material.
